# Whole Blood Gene Expression Testing for Coronary Artery Disease in Nondiabetic Patients: Major Adverse Cardiovascular Events and Interventions in the PREDICT Trial

**DOI:** 10.1007/s12265-012-9353-z

**Published:** 2012-03-07

**Authors:** Steven Rosenberg, Michael R. Elashoff, Hsiao D. Lieu, Bradley O. Brown, William E. Kraus, Robert S. Schwartz, Szilard Voros, Stephen G. Ellis, Ron Waksman, John A. McPherson, Alexandra J. Lansky, Eric J. Topol, for the PREDICT Investigators

**Affiliations:** 1 CardioDx, Inc., 2500 Faber Place, Palo Alto, CA 94303 USA; 2 Duke Center for Living, 3475 Erwin Road, Box 3022, Room 254, Aesthetics Building, Durham, NC 27705 USA; 3 Minneapolis Heart Institute Foundation, Abbott Northwestern Hospital, 920 East 28th Street, Suite 620, Minneapolis, MN 55407 USA; 4 Piedmont Heart Institute, 95 Collier Road NW, Suite 2035, Atlanta, GA 30309 USA; 5 The Cleveland Clinic, 9500 Euclid Avenue, F25, Cleveland, OH 44195 USA; 6 Cardiovascular Research Institute, Medstar Research Institute, Washington Hospital Center, 110 Irving Street NW, Suite 6B-5, Washington, DC 20010 USA; 7 Vanderbilt University, 1215 21st Avenue South, MCE 5th Floor, South Tower, Nashville, TN 37232 USA; 8 Yale University School of Medicine, Fitkin Pavilion, New Haven, CT 06520 USA; 9 Scripps Translational Science Institute, 3344 North Torrey Pines Court, La Jolla, CA 92037 USA

**Keywords:** Coronary artery disease, Peripheral blood gene expression, Genomics, Angiography, Coronary interventions, MACE

## Abstract

The majority of first-time angiography patients are without obstructive coronary artery disease (CAD). A blood gene expression score (GES) for obstructive CAD likelihood was validated in the PREDICT study, but its relation to major adverse cardiovascular events (MACE) and revascularization was not assessed. Patients (*N* = 1,160) were followed up for MACE and revascularization 1 year post-index angiography and GES, with 1,116 completing follow-up. The 30-day event rate was 23% and a further 2.2% at 12 months. The GES was associated with MACE/revascularizations (*p* < 0.001) and added to clinical risk scores. Patients with GES >15 trended towards increased >30 days MACE/revascularization likelihood (odds ratio = 2.59, 95% confidence interval = 0.89–9.14, *p* = 0.082). MACE incidence overall was 1.5% (17 of 1,116) and 3 of 17 patients had GES ≤15. For the total low GES group (*N* = 396), negative predictive value was 90% for MACE/revascularization and >99% for MACE alone, identifying a group of patients without obstructive CAD and highly unlikely to suffer MACE within 12 months.

**Electronic supplementary material:**

The online version of this article (doi:10.1007/s12265-012-9353-z) contains supplementary material, which is available to authorized users.

## Introduction

Chronic coronary artery disease (CAD) and adverse cardiovascular events are the largest sources of morbidity and mortality in the developed world and are diagnosed in more than 500,000 new patients annually in the USA [1]. Obstructive CAD diagnosis is challenging as patient presentation may often be variable and atypical symptoms are common [2]. Clinical evaluation of suspected CAD often includes stress testing followed by noninvasive imaging (stress echocardiography or nuclear perfusion) and, if indicated, invasive coronary angiography. Recent studies have highlighted the relatively high radiation exposure burden in the standard CAD workup [3, 4] and have indicated that, for patients without a prior CAD diagnosis, <40% have obstructive CAD when referred for coronary angiography [5]. In addition, the COURAGE trial suggested that optimal medical therapy was noninferior to percutaneous coronary intervention (PCI) for hard cardiovascular endpoints in patient populations with stable angina and CAD [6]. Thus, noninvasive genomic-based methods for CAD diagnosis may have significant clinical utility and lead to lower diagnostic costs in these patient populations.

We described differential blood cell gene expression levels in patients with CAD [7] and, more recently, the development and clinical validation in the PREDICT study of a gene expression score (GES) comprised of the expression levels of 23 genes, age, and sex [8, 9]. In this angiographic population of nondiabetic patients, approximately 80% were symptomatic and the quantitative coronary angiography (QCA)-defined obstructive CAD prevalence was 36%; the GES negative predictive value (NPV) was 83% at a score threshold of 15, with 33% of patients below this threshold. Furthermore, the GES correlated with QCA-determined maximum percent stenosis. To evaluate the outcomes of these GES patients, we monitored 1,160 PREDICT patients for major adverse cardiovascular events (MACE) and interventional procedures for 12 months from index catheterization.

## Methods

### General Study Design and Study Population

Subjects were enrolled in PREDICT, a 39-center prospective study, between July 2007 and April 2009 (http://www.clinicaltrials.gov, NCT 00500617). The study complied with the Declaration of Helsinki, was approved by institutional review boards at all centers, and all patients gave written informed consent. Subjects referred for diagnostic coronary angiography were eligible with a history of chest pain, suspected angina equivalent symptoms, or a high risk of CAD and no known prior myocardial infarction (MI), revascularization, or obstructive CAD. Subjects were ineligible if at catheterization they had acute MI, high-risk unstable angina, severe noncoronary heart disease (congestive heart failure, cardiomyopathy, or valve disease), systemic infectious or inflammatory conditions, or were taking immunosuppressive or chemotherapeutic agents. Detailed eligibility criteria have been described [9].

From 1,354 enrolled nondiabetic subjects who met the inclusion criteria, 5 had angiographic images unsuitable for QCA and 6 had unusable blood samples. The remaining 1,343 were divided into independent algorithm development and validation cohorts sequentially based on enrollment [9]; of these, 1,166 patients had valid GES, 640 in algorithm development and 526 in validation [9]. These were evaluated for events, with six subjects from algorithm development not meeting the clinical inclusion criteria upon further evaluation.

### Clinical Evaluation and Quantitative Coronary Angiography

Prespecified clinical data, including demographics, medications, clinical history, and presentation, were obtained by research study coordinators using standardized data collection methods and verified by independent study monitors.

Coronary angiograms were analyzed by computer-assisted QCA. Specifically, clinically indicated coronary angiograms performed according to site protocols were digitized, deidentified, and analyzed with a validated quantitative protocol at the Cardiovascular Research Foundation, New York, NY, USA [10]. All lesions >10% diameter stenosis (DS) in vessels with diameter >1.5 mm were visually identified, and the minimal lumen diameter (MLD), reference lumen diameter (RLD = average diameter of normal segments proximal and distal of lesion), and %DS (%DS = (1 − MLD/RLD) × 100) were calculated.

The Diamond–Forrester (D-F) risk score, comprised of age, sex, and chest pain type, was prospectively chosen to evaluate the value of the GES with clinical factors [11]. D-F classifications of chest pain type (typical angina, atypical angina, and nonanginal chest pain) were assessed using subject interviews [11] and D-F scores assigned [12].

### Obstructive CAD and Disease Group Definitions

Obstructive CAD (*N* = 422) was defined prospectively as ≥1 atherosclerotic plaque in a major coronary artery (≥1.5 mm lumen diameter) causing ≥50% luminal DS by QCA; nonobstructive CAD (*N* = 744) had no lesions >50%.

### Clinical Procedure and Event Determination

Clinical interventions were defined as any PCI or coronary artery bypass graft (CABG). Clinical events were defined as stroke/transient ischemia attack (TIA), MI, or death. Index coronary angiography was defined as the date of planned coronary catheterization, irrespective of intervention. Coronary procedures or events occurring within 30 days of index angiography were considered baseline endpoints associated with this procedure. In addition, specifically identified staged procedures up to 45 days post-index angiography were also considered baseline endpoints. Analysis of all procedures and events was performed for the 1,160 subjects over the entire follow-up period, as well as selective analysis for patients with procedures and events beyond the 30-day threshold.

All coronary procedures and events were monitored against medical records for accuracy and were supported by medical records documenting the specific event or diagnosis and/or by supporting evidence, e.g., myocardial enzyme elevation or infarct on head computed tomography (CT). Discrepancies were resolved by direct investigator query. All other events such as aortic aneurysm repair, congestive heart failure exacerbation, and cardiac arrhythmias were reviewed and eliminated due to noncardiac origin or lack of direct association with acute coronary atherosclerosis etiology. The definitions of the MACE components, MI, stroke/TIA, and all-cause mortality are detailed in the Supplementary Methods.

### GES Measurements

GES measurements were performed in the CardioDx clinical reference laboratory (Palo Alto, CA, USA) using the Corus™ CAD process [9]. Briefly, RNA was purified using an automated bead-based method from PAXgene® RNA preservation tubes (PreAnalytiX, Valencia, CA, USA). Subsequent cDNA synthesis and reverse transcription polymerase chain reaction were then carried out [9]. The GES were reported on a 1–40 scale.

### Statistical Analysis

The primary endpoint for the study was whether the GES as a continuous variable was significantly related to the combination of procedures and MACE at 30 days and 12 months following index angiography. Subjects were censored if no event occurred prior to them being lost to follow-up. Only the first endpoint of a given type (procedure or event) was counted in the analysis. Secondary analyses included the relationship of the GES to MACE across the entire follow-up period and to the combination of revascularizations and MACE occurring >30 post-index catheterization.

For categorical analyses, the GES were divided into three ranges: 1–15 (<20% likelihood), 16–27 (≥20–<50% likelihood), and 28–40 (≥50% likelihood) [9]. Logistic regression was used to test the relation between the GES (continuous) and events/procedures; for comparison to clinical factor scores, multivariate logistic regression was used. Odds ratios (OR), associated 95% confidence intervals (95% CI), and *p* values were also estimated by logistic regression. A prespecified GES threshold of ≤15 was used to estimate test performance (sensitivity, specificity, NPV, and positive predictive value [PPV]), as well as for categorical GES OR analyses. The Cochran–Armitage trend test was used to test the relation between GES categories and events/procedures. Clinical factors were compared at baseline using either a two-sample *t* test (continuous measures) or Fisher’s exact test (binary measures). All analyses were performed in R version 2.11 [13].

## Results

From 1,166 sequential PREDICT patients comprising the algorithm development and clinical validation cohorts with QCA and GES [9], 1,160 were eligible for follow-up and 1,116 (96%) were followed up for 1 year after index angiography. Clinical and angiographic characteristics of this entire cohort and the clinical validation subset (*N* = 526) are shown in Table 1. The entire cohort was 58% male with an average age of 60. Factors which were significantly (*p* < 0.001) associated with angiographically defined obstructive CAD at baseline included male sex, age, systolic blood pressure (SBP), dyslipidemia, smoking status, chest pain symptoms, higher body mass index (BMI), aspirin, statin, and beta-blocker use (Table 1). Only 36% of patients had obstructive CAD (≥50% maximum percent stenosis) at index angiography.

**Table 1:** Clinical and demographic characteristics of PREDICT patient cohorts

Set parameter	Complete cohort	Clinical validation subset
*N*	1,160	526
Male sex	668 (57.6%)	299 (56.8%)
Age	59.9 ± 11.8 (25.5 to 90.9)	60.3 ± 11.6 (25.6 to 90.9)
SBP	134.9 ± 18.3 (88 to 213)	135.2 ± 18.4 (90 to 213)
Dyslipidemia	734 (63.3%)	341 (64.8%)
Smoker	412 (35.5%)	186 (35.4%)
Symptomatic	762 (65.7%)	359 (68.3%)
BMI	30.8 ± 6.8 (13.8 to 69.4)	30.7 ± 6.5 (13.8 to 61.7)
Aspirin use	768 (66.5%)	363 (69.1%)
Statin use	580 (50.2%)	265 (50.5%)
Beta-blocker use	425 (36.8%)	212 (40.4%)
QCAMaxStenosis^a^	38.2 ± 32.3 (0 to 100)	38.9 ± 32.1 (0 to 100)
QCANumLesions^b^	1.8 ± 2.3 (0 to 12)	1.9 ± 2.4 (0 to 10)
QCAObsDisease^c^	420 (36.2%)	192 (36.5%)
One-year follow-up	1,115 (96.1%)	507 (96.4%)

^a^Maximum percent stenosis determined by core laboratory QCA

^b^Number of ≥30% stenotic lesions by QCA

^c^Patients with ≥50% stenosis in a major coronary artery by QCA

**Fig. 1:**
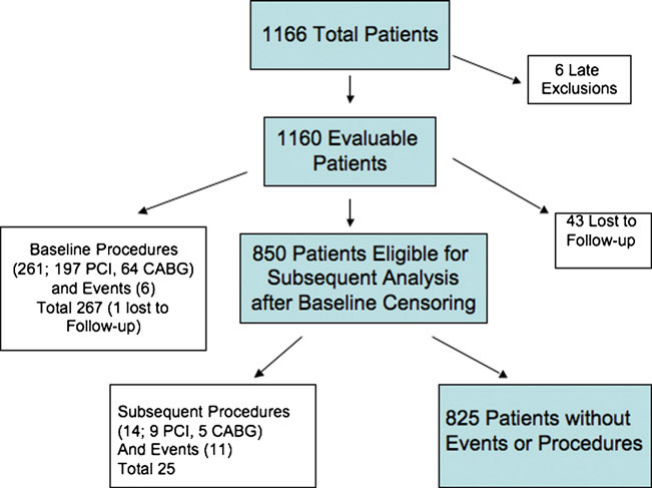
Schematic of patient flow and endpoint summary. A total of 1,166 patients from the algorithm development and validation cohorts were followed up. There were 6 late clinical exclusions, resulting in a final cohort of 1,160 of whom follow-up data was available for 1,143 (96%). A total of 267 had interventional procedures or events associated with their index angiographic procedure (within 30 days). The remaining 850 patients had a total of 25 endpoints (14 interventional procedures and 11 adverse events) in the subsequent follow-up period, for a total of 292 endpoints (25%) over 1 year

The patient study flow is shown schematically in Fig. 1. A total of 267 patients (23%) had endpoints within 30 days of index procedure with the vast majority being PCI or CABG. After censoring these patients, there were only 25 additional patients (3%) with procedures or events in the next year out of the remaining 850, yielding an overall endpoint rate of 25% for all patients in the entire period. For MACE alone, the rate was 1.5% for 12 months. Events and procedures are summarized in Table 2.

**Table 2:** Summary of procedures and events at 1-year follow-up

Parameter	
*N* at index angiogram	1,160
Baseline procedures	
PCI	203 (17.5%)
CABG	70 (6%)
Total procedures^a^	267 (23%)
Baseline events	6 (0.5%)
All baseline endpoints	267 (23%)
*N* with follow-up	1,116 (96%)
Follow-up procedures	14 (1.2%)
Follow-up events	11 (0.9%)
Total follow-up endpoints	25 (2.2%)
All procedures	286 (24.7%)
All events	17 (1.5%)
All endpoints^b^	292 (25.2%)

^a^Some patients had more than one procedure and four patients had events after baseline procedures

^b^The total baseline number of patients is used as the denominator for all calculations as baseline endpoints greatly dominate total endpoints. Some patients had more than one endpoint

### GES Analysis

**Fig. 2:**
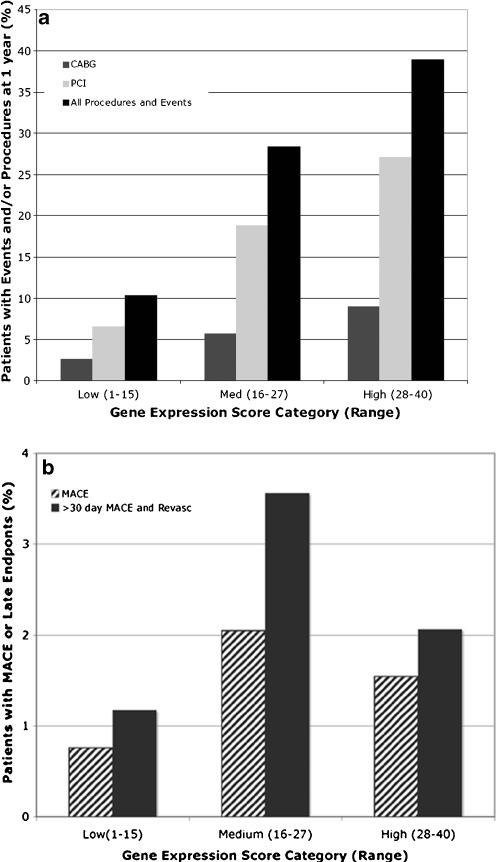
**a** Dependence of event and interventional procedure likelihood on GES in 1 year. The percentage of patients who had interventional procedures or events within 1 year of the index catheterization are shown stratified by GES. GES are divided into low (1–15), medium (16–27), and high (28–40) categories as described in the text. Results are shown for the entire cohort of 1,160 patients. **b** Dependence of MACE likelihood on GES in 1 year. The percentage of patients who had MACE within 1 year of index catheterization are shown stratified by GES (*striped bars*). The percentage of patients with revascularization or MACE >30 days post-index catheterization are shown stratified by GES (*solid bars*). Scores are divided as in **a**. There were 3, 9, and 5 events for MACE alone (*striped bars*) and 4, 11, and 4 revascularizations and MACE (*solid bars*) in the low, medium, and high GES categories

The GES, comprised of the peripheral blood cell expression levels of 23 genes and sex-specific age dependencies of CAD likelihood, was associated with the composite primary endpoint of MACE and procedures over 1 year by logistic regression (*p* < 0.001) and added to clinical factors, as quantified by D-F or Framingham risk scores (Supplementary Table 1). GES category also correlated with the likelihood of the combined procedures and MACE primary endpoint over this period as shown in Fig. 2a.

Previous analysis of obstructive CAD in the PREDICT clinical validation study identified a low likelihood (<20%) group with GES ≤15 [9]. Using this threshold for the primary composite endpoint at 12 months follow-up, the sensitivity and specificity were 86% and 41%, respectively, corresponding to the NPV of 90% and PPV of 33%, with 396 patients (35%) in this group (Table 3). The OR for those with nonlow scores (>15) versus low scores (≤15) for the 30-day and 12-month endpoints were 4.3 (95% CI, 3.0–6.4) and 4.3 (95% CI, 3.0–6.3), respectively, both *p* < 0.001 (Table 3).

**Table 3:** Dependence of combined procedure and MACE risk on GES

Duration and endpoints	NPV^a^ (%)	PPV (%)	Sensitivity (%)	Specificity (%)	OR^b^	95% CI	*P* value
12-month procedures and MACE^c^	90	33	86	41	4.32	3.02–6.25	<0.001
12-month MACE	99	1.8	82	34	2.41	0.74–10.5	0.16^d^
≤30-day procedures and MACE^e^	91	33	87	40	4.31	3.00–6.38	<0.001
>30-day procedures and MACE	99	3.0	79	41	2.59	0.89–9.14	0.082^e^

^a^NPV, PPV, sensitivity, and specificity were calculated at a threshold of 15

^b^OR were calculated with a GES threshold of 15

^c^Procedures (PCI or CABG) and MACE (MI, stroke/TIA, death) within 12 months of the index angiography

^d^Not significant

^e^Procedures and MACE occurring within 30 days of the index angiography

There were 17 patients with MACE, of which 15 occurred more than 30 days after index angiogram; 4 of these patients had early revascularization. The clinical, angiographic, and MACE characteristics for all patient events are summarized in Table 4. The GES at index procedure was above 15 in 14 of 17 of these patients (Table 4, Fig. 2b). Thus, at most, 3 patients of 1,160 (0.3%) had both a low GES and an adverse event in the following year, yielding an NPV for events alone of 99.2%, although this did not reach statistical significance (OR = 2.41, 95% CI = 0.74–10.4, *p* = 0.16). There were a total of eight patients with late revascularizations whose characteristics are summarized in Table 5, with seven of eight having GES above 15. Patients with either late revascularizations or MACE more than 30 days post-index catheterization trended towards higher GES (OR = 2.59, 95% CI, 0.89–9.14, *p* = 0.082) (Table 3); the relationship between the GES and these late revascularizations and events are illustrated in Fig. 2b.

**Table 4:** Clinical characteristics of patients with subsequent events

Patient ID	Sex	Age (years)	QCACase:Control^a^	QCAMax Stenosis	ClinMax Stenosis	QCANum Lesions30^b^	GES	Event	Days post index
C002:00400185	Male	83	Control	0	15	0	31	Stroke or TIA	328
C003:00400346	Female	58	Case	70	0	1	10	MI^c^	121
C004:00400011	Female	73	Control	39	50	1	17	MI	259
C005:00400009	Male	50	Case	100	100	1	18	CABG, MI, PCI	10
C014:00400055	Male	60	Case	76	90	5	29	Stroke or TIA	566^d^
C015:00400040	Male	51	Case	57	40	3	24	Stroke or TIA	>180^d^
C015:00400058	Female	46	Control	0	0	0	2	MI^e^	339
C015:00400064	Male	66	Control	15	80	0	25	Stroke or TIA	321
C015:00400092	Male	49	Control	19	30	0	25	MI	>180^f^
C015:00400193	Female	66	Case	78	70	6	16	MI	129
C051:00400030	Male	63	Case	75	90	6	26	MI	1
C058:00400054	Male	69	Control	33	40	2	25	Death	>180^f^
C063:00400007	Female	76	Case	80	90	5	27	MI	177^d^
C068:00400065	Male	86	Case	81	95	1	37	Stroke or TIA	235^d^
C073:00400040	Male	73	Control	44	65	1	30	Stroke or TIA	172^d^
C073:00400065	Male	60	Case	63	40	3	14	MI	224
C079:00400014	Male	78	Case	100	50	7	39	Death	306

^a^Prospectively defined as ≥50% maximum stenosis

^b^Number of lesions >30% stenosis by QCA

^c^Discrepancy between clinical and core laboratory QCA reads; QCA confirmed on subsequent independent review

^d^These patients had a revascularization associated with their index catheterization

^e^Likely vasospastic MI given underlying clinical condition and chart review

^f^Event reported at 1 year follow-up without specific date

**Table 5:** Clinical characteristics of patients with late revascularizations

Patient ID	Sex	Age (years)	QCACase:Control^a^	QCAMax Stenosis	ClinMax Stenosis	QCANum Lesions30^b^	GES	Procedure^c^	Days post index
C015:00400017	Male	54.4	Case	60.37	70	3	26	PCI	341
C054:00400009	Female	70.3	Case	100	80	3	25	CABG	75
C015:00400060	Male	55.5	Control	36.43	100	3	23	PCI	118
C055:00400036	Female	68.3	Control	24.2	40	0	20	PCI	345
C068:00400058	Female	55.2	Case	100	99	8	3	PCI	347
C001:00400105	Male	73.7	Case	60.7	90	2	32	PCI	70
C015:00400177	Male	64.5	Control	43.19	50	2	26	PCI	246
C068:00400087	Male	68.1	Case	100	100	5	37	CABG	84

^a^Prospectively defined as ≥50% maximum stenosis

^b^Number of lesions >30% stenosis by QCA

^c^Either PCI or CABG occurring without prior intervention associated with index catheterization

## Discussion

This study extended our previous validation of a blood-based GES for obstructive CAD in nondiabetic patients from an angiographic endpoint to revascularizations and MACE. We followed up and identified revascularizations and adverse events in 1,160 patients from the PREDICT trial, including the previously defined validation cohort of 526 patients for 12 months from index procedure. As expected, revascularization (PCI and CABG) were closely associated with maximum percent stenosis and angiographically determined disease burden, with the exception of chronic total occlusions which had a reduced intervention rate.

Our previous analysis showed that, in the validation set of 526 patients, using obstructive CAD as the endpoint, 33% of patients had GES ≤15 with an NPV of 83%. For actual clinical endpoints up to 1 year, the NPV for all procedures and MACE was 90% at this threshold in the entire cohort. For those patients with GES ≤15 (396 of 1,160), representing 35% of total enrollment, only 41 of 1,160 (3.5%) had procedures or events. In these patients, the majority of endpoints (28 of 41) were PCI which has not been shown to improve long-term outcomes over optimal medical therapy in the COURAGE population [6].

It has been demonstrated that the fraction of obstructive CAD at cardiac catheterization in US patients without known CAD is 35–40% [5, 9]. In the entire cohort in this study, the yield of obstructive CAD was 36.2% and the fraction of patients with interventions was 23.7%. If one did not send patients with low GES for catheterization, the yield of patients with obstructive CAD and interventions would be increased to 48.2% and 31%, respectively.

We previously observed that increasing GES correlated with maximum percent stenosis. In the current analysis, the composite endpoint likelihood also monotonically increased with GES from approximately 10% for low scores to >35% with high scores (28–40) with an OR of >4 (Fig. 2a). For high scores, this was likely an underestimate as >80% of patients with chronic total occlusions, who were electively not intervened on, had high scores. A large recent study of patients referred for CT angiography has also shown that overall mortality risk correlated with the extent of maximum percent stenosis [14].

For the small number of patients who had events, >80% (14 of 17) had GES above the threshold of 15 (Tables 3 and 4), although this did not reach statistical significance. Retrospective analysis for the three patients with events and low GES showed one patient had no CAD angiographically with a GES of 2 and likely suffered a vasospastic MI. A second patient had no CAD by clinical angiogram, but subsequent QCA showed a 70% lesion. The third patient had a score of 14, close to the threshold, and an MI 7 months from index procedure. Thus, based upon clinical workup, 16 of 17 patients with events had scores above the threshold. Similarly, for late revascularizations, seven of eight had scores above 15.

A description of the genes which comprise the GES are shown in Table 6, along with the associated biological functions, where known. The predominant features of these gene terms are the innate immune response, as judged by increased expression of activation genes in both neutrophils and natural killer (NK) cells, as well as an increase in proapoptotic genes (terms 1–3). In addition, term 2, and specifically S100A12, has been shown to promote coronary artery calcification in a transgenic model [15]. In addition, the somewhat counterintuitive B cell to T cell ratio comprises term 4. Although it was originally thought that B cells were atheroprotective and T cells atherogenic, recent work in mouse models has suggested a more complex picture with atherogenic B cell subsets [16] and a potential atheroprotective role for regulatory T cells [17, 18].

**Table 6:** GES components and putative biological roles

Term	Genes	Functions
1	IL18RAP+TNFAIP6+CASP5	Innate immunity, apoptosis
	IL8RB+KCNE3+TLR4+TNFRSF10C	Neutrophil activation
2	S100A8+S100A12+CLEC4E	Neutrophil activation and necrosis
	RPL28 (men), NCF4+AQP9 (women)	Calcification
		Neutrophil/lymphocyte ratio (men)
		Normalized neutrophil activation (women)
3	SLAMF7+KLRC4	Innate immunity, NK cell activation
	TMC8+CD3D	Normalized to T lymphocytes
4	SPIB+CD79B	B/T cell ratio
	TMC8+CD3D	Lymphocyte subtype
5 + 6	AF289562+TSPAN16 (men)	Unknown function genes
	TFCP2+HNRPF	

Given that the GES was derived to discriminate obstructive CAD, why might it have prognostic value? First, the GES is proportional to maximum percent stenosis by angiography and a recent large CT angiography study has shown that event likelihood increases with the extent of disease, even for nonobstructive disease [19]. Second, specific terms in the GES algorithm reflect cell type-specific gene expression ratios, which in the case of the neutrophil to lymphocyte ratio has been shown to have prognostic significance in a large catheterization laboratory population [20]. In addition, a very recent large study has shown that neutrophil counts alone are associated with subsequent MI and mortality [21]. Third, circulating levels of the protein products of genes which are present in the GES, such as S100A8 and S100A12, have been shown to be associated with cardiovascular events [22, 23]. Finally, the observed GES proportionality to disease burden is most likely a reflection of the dysregulation of gene expression in the circulating cells in response to both the extent and inflammatory activity of atherosclerotic plaque, perhaps reflecting plaque composition.

This study had several limitations. First, the population was nondiabetic and largely symptomatic with high-risk unstable angina and low-risk asymptomatic patients excluded. Second, the follow-up period was limited and the number of events subsequent to the index catheterization small. Thus, any conclusions about the PPV of the GES for prognosis will require larger cohorts, more extended follow-up, and a higher absolute number of cumulative events. Given the observed OR for MACE in this study, we estimate that a study of 2,300 patients with 2-year follow-up would have 80% power to detect a significant relationship of the GES to MACE. The PROMISE study (http://www.clinicaltrials.gov, NCT 01174550) might be an appropriate setting to further test this hypothesis. Third, we did not have lesion-specific information to determine if revascularizations or events were due to baseline-identified lesions or disease progression. Fourth, since this was an angiographic population, it had more disease than an intended use population before referral, which may affect the results. Fifth, with respect to the GES analysis, the combined cohort may have been biased by inclusion of the algorithm development set. This seems unlikely to be a very significant factor as procedures and events were not used to derive the algorithm, and the validation subset analyses showed results indistinguishable from the entire population. Finally, while the GES added significantly to Framingham with respect to the primary composite endpoint, it did not add significantly to MACE prediction alone, although that comparison was underpowered due to the low event rate.

In summary, this study examined the relationship between a peripheral blood GES measured at index angiography and revascularization and MACE at up to 12 months. Independent of the GES, more than 75% of patients had neither a procedure nor MACE in the next year. For those with low GES, representing 35% of patients, 90% were in this category. Thus, low GES appeared to identify a population at low risk for both obstructive CAD and subsequent procedures or events. While these results were encouraging for a clinical correlation with the initial angiographic validation, studies in larger populations with longer-term follow-up would be needed to further support this hypothesis.

### Electronic Supplementary Material

Below is the link to the electronic supplementary material.

ESM 1 (DOC 66 kb)

## Appendix 1: PREDICT Site Principal Investigators

Naeem Tahirkheli, Oklahoma Cardiovascular Research Group, Oklahoma City, OK, USA; Daniel Donovan, Cardiology Clinic of San Antonio, San Antonio, TX, USA; Stanley Watkins, Alaska Heart Institute, Anchorage, AK, USA; Brian Beanblossom, Cardiovascular Associates, Louisville, KY, USA; Brent Muhlestein, Intermountain Health Care, Salt Lake City, UT, USA; Ronald Blonder, Pikes Peak Cardiology, Colorado Springs, CO, USA; Tim Fischell, Borgess Research Medical Center, Kalamazoo, MI, USA; Phillip Horwitz, University of Iowa Hospitals, Iowa City, IA, USA; Frank McGrew, The Stern Cardiovascular Center, Germantown, TN, USA; Tony Farah, Allegheny Professional Building, Pittsburgh, PA, USA; Terrance Connelly, Charlotte Heart Group Research Center, Port Charlotte, FL, USA; Cezar Staniloae, New York Cardiovascular Assoc./Heart and Vascular Research, New York, NY, USA; Edward Kosinski, Connecticut Clinical Research, LLC, Bridgeport, CT, USA; Charles Lambert, University Community Health, Tampa, FL, USA; David Hinchman, St Luke’s Idaho Cardiology Associates, Boise, ID, USA; James Zebrack, Heart Center, Salt Lake City, UT, USA; Bruce Samuels, Cardiovascular Medical Group of Southern California, Beverly Hills, CA, USA; Matthew Budoff, Los Angeles Biomedical Research Institute at Harbor–University of California Los Angeles Medical Center, Torrance, CA, USA; Dean Kereiakes, The Lindner Clinical Trial Center, Cincinnati, OH, USA; Christopher Brown, Mobile Heart Specialists, Mobile, AL, USA; Jennifer Hillstrom, Maine Cardiology, Portland, ME, USA; Donald Wood, Peninsula Cardiology Associates, Salisbury, MD, USA; Hossein Amirani, Via Christi Research, Wichita, KS,USA; Jeffrey Bruss, Hoag Heart and Vascular Institute, Newport Beach, CA, USA; Ronald Domescek, Orlando Heart Center, Orlando, FL, USA; Stephen Burstein, Los Angeles Cardiology Associates, Los Angeles, CA, USA; Mark Heckel, Carolina Heart Specialists, Gastonia, NC, USA; Barry Clemson, Heartcare Midwest SC, Peoria, IL, USA; Charles Treasure, Cardiovascular Research Foundation, Knoxville, TN, USA; Ricky Schneider, Cardiology Consultants of South Florida, Tamarac, FL, USA; Hassan Ibrahim, North Ohio Heart Center, Sandusky, OH, USA; Robert Weiss, Maine Research Associates, Auburn, ME, USA; John Eagan Jr., Office of Clinical Research, Birmingham, AL, USA; David Henderson, Cardiology Research Associates, Ormond Beach, FL, USA; Lev Khitin, Chicago Heart Institute, Elk Grove, IL, USA; and Preet Randhawa, New Jersey Heart Research, Linden, NJ, USA.

## Abbreviations

GES: Gene expression score
CAD: Coronary artery disease
MACE: Major adverse cardiovascular events
NPV: Negative predictive value
MI: Myocardial infarction
QCA: Quantitative coronary angiography
D-F: Diamond–Forrester score
PCI: Percutaneous coronary intervention
CABG: Coronary artery bypass graft
TIA: Transient ischemia attack
