# Supplementary material for: Whole Blood Gene Expression Testing for Coronary Artery Disease in Nondiabetic Patients: Major Adverse Cardiovascular Events and Interventions in the PREDICT Trial
Source: J Cardiovasc Transl Res. 2012 Mar 7;5(3):366–74. doi: 10.1007/s12265-012-9353-z (PMC3349850; doi:10.1007/s12265-012-9353-z)
Supplement: Supplementary file 1 — (DOC 66 kb) [file 12265_2012_9353_MOESM1_ESM.doc]

**Supplementary Materials**

Supplementary Methods – Detailed Definitions of the Components of MACE for this Study

**Myocardial Infarction (MI)**

Current myocardial infarction is defined by abnormally elevated cardiac markers associated with an appropriate clinical syndrome. Current is defined as occurring during the index workup (defined below).

§         *ST Elevation Myocardial Infarction (STEMI)*

Myocardial infarction associated with at least 1mm ST segment elevation above baseline in at least two contiguous electrocardiographic leads

§         *Non-ST Elevation Myocardial Infarction (NSTEMI)*

Myocardial infarction not meeting the criteria for STEMI

**All Cause Mortality (Death)**

*Cardiac death* is defined as any death in which a cardiac cause cannot be excluded. (This includes but is not limited to acute myocardial infarction, cardiac perforation/pericardial tamponade, arrhythmia or conduction abnormality,  cerebrovascular accident suspected of being related to a cardiac procedure, death due to complication of the procedure, including bleeding, vascular repair, transfusion reaction, or bypass surgery.)

*Non-cardiac death* is defined as a death not due to cardiac causes (as defined above).

**Stroke or Cerebrovascular Accident (CVA)**

The occurrence of cerebral infarction (ischemic stroke) and intracerebral hemorrhage and subarachnoid hemorrhage (hemorrhagic stroke).

**Transient Ischemic Attack (TIA)**

A brief episode of neurologic dysfunction caused by focal brain or retinal ischemia, with clinical symptoms typically lasting less than 1 hour, and without evidence of infarction.

Supplementary Table 1. Multivariate Logistic Regression Analysis of Gene Expression and Clinical Risk Scores for Endpoints.

| Patient Set | Classifier | Odds Ratio | p-value |
| --- | --- | --- | --- |
| All | Diamond-Forrester^1^  Gene Expression^2^ | 1.014  1.058 | <0.001  <0.001 |
| All | Framingham^3^  Gene Expression | 1.046  1.047 | <0.001  <0.001 |
| Validation Subset | Diamond-Forrester  Gene Expression | 1.018  1.047 | <0.001  <0.001 |
| Validation Subset | Framingham  Gene Expression | 1.048  1.040 | 0.003  0.009 |

^1^ Odds Ratios for Diamond-Forrester per unit % likelihood of obstructive disease (0-100)

^2^ Odds Ratios for Gene Expression per unit score (0-40 scale)

^3^ Odds Ratios for Framingham Risk Score per unit % 10 year likelihood (0-30 scale
